# Comparison of health information exchange data with self-report in measuring cancer screening

**DOI:** 10.1186/s12874-023-01907-7

**Published:** 2023-07-25

**Authors:** Oindrila Bhattacharyya, Susan M. Rawl, Stephanie L. Dickinson, David A. Haggstrom

**Affiliations:** 1 Department of Economics, Indiana University Purdue University, Indianapolis, IN USA; 2 James Comprehensive Cancer Center, The Ohio State University, Columbus, OH USA; 3 The William Tierney Center for Health Services Research, Regenstrief Institute Inc, Indianapolis, IN USA; 4 Indiana University School of Nursing, Indiana University Melvin and Bren Simon Cancer Comprehensive Center, Indianapolis, IN USA; 5 Department of Epidemiology and Biostatistics, Indiana University School of Public Health-Bloomington, Bloomington, IN USA; 6 VA HSR&D Center for Health Information and Communication, Roudebush VA, Indianapolis, IN USA; 7 Division of General Internal Medicine and Geriatrics, Indiana University School of Medicine, Indianapolis, IN USA; 8 Center for Health Services Research, Regenstrief Institute, Indianapolis, IN USA; 9 Indiana University Cancer Center, Indianapolis, IN USA

**Keywords:** Early detection, Cancer screening, Survey, Electronic medical records

## Abstract

**Background:**

Efficient measurement of the receipt of cancer screening has been attempted with electronic health records (EHRs), but EHRs are commonly implemented within a single health care setting. However, health information exchange (HIE) includes EHR data from multiple health care systems and settings, thereby providing a more population-based measurement approach. In this study, we set out to understand the value of statewide HIE data in comparison to survey self-report (SR) to measure population-based cancer screening.

**Methods:**

A statewide survey was conducted among residents in Indiana who had been seen at an ambulatory or inpatient clinical setting in the past year. Measured cancer screening tests included colonoscopy and fecal immunochemical test (FIT) for colorectal cancer, human papilloma virus (HPV) and Pap tests for cervical cancer, and mammogram for breast cancer. For each screening test, the self-reported response for receipt of the screening (yes/no) and ‘time since last screening’ were compared with the corresponding information from patient HIE to evaluate the concordance between the two measures.

**Results:**

Gwet’s AC for HIE and self-report of screening receipt ranged from 0.24–0.73, indicating a fair to substantial concordance. For the time since receipt of last screening test, the Gwet’s AC ranged from 0.21–0.90, indicating fair to almost perfect concordance. In comparison with SR data, HIE data provided relatively more additional information about laboratory-based tests: FIT (19% HIE alone vs. 4% SR alone) and HPV tests (27% HIE alone vs. 12% SR alone) and less additional information about procedures: colonoscopy (8% HIE alone vs. 23% SR alone), Pap test (13% HIE alone vs. 19% SR alone), or mammography (9% HIE alone vs. 10% SR alone).

**Conclusion:**

Studies that use a single data source should consider the type of cancer screening test to choose the optimal data collection method. HIE and self-report both provided unique information in measuring cancer screening, and the most robust measurement approach involves collecting screening information from both HIE and patient self-report.

**Supplementary Information:**

The online version contains supplementary material available at 10.1186/s12874-023-01907-7.

## Introduction

Evidence-based screening tests are important for early detection of cancer to reduce the likelihood of a more advanced stage at diagnosis when cancer may be less treatable [1]. Information about the receipt of cancer screening tests is widely available from patient data stored in electronic health records (EHR), a systematic collection of patient health information in a digital format [2]. Another alternative source of information about cancer screening is from patients’ self-report, an arguably more cost-effective source [3]. EHRs may benefit both clinical and epidemiologic research, patient care, as well as performance measurement. However, structured EHR data inevitably contains incomplete or inaccurate information. Furthermore, EHRs typically are deployed in a single health care practice, hospital, or system. Health information exchange (HIE), which involves the electronic exchange of clinical and administrative information across a variety of health care organizations, provides a more population-based approach to the measurement of cancer screening as it contains EHR data from multiple health care settings or systems [4, 5].

To our knowledge, no prior statewide studies of cancer screening has compared HIE data with patient self-report. Also, previous literature comparing self-report and EHR data showed varying levels of agreement based on factors such as type of clinical condition, type of clinical procedures performed, or data collection methods [6–10]. Given the varying results of previous validation studies and the implications for population-based measurement in the use of HIE, we assessed the concordance between self-reported cancer screening by survey among Indiana residents and the individuals’ corresponding information from the statewide Indiana Health Information Exchange (IHIE). We focused on the cancer screening phase of the cancer care continuum for colorectal, cervical, and breast cancers. We hypothesized that the value of information gained from different data sources on cancer screening, might vary according to the types of screening tests.

## Methods

From January 2018 through February 2018, Indiana residents who had been seen at least once in the previous year at Indiana University Health (IU Health) participated in a cross-sectional, mail-based survey known as the Hoosier Health Survey. A statewide integrated health system, IU Health operates 16 hospitals in Indiana and 178 clinics that provide outpatient care; it is the largest health system in the state, had 115,690 admissions in 2021 with a leading 31.3% market share in its primary service area (PSA) in central Indiana [11]. The purpose of this survey was to better understand the cancer control needs of the community served by the Indiana University Cancer Center [12]. The population catchment area of IU Cancer Center as defined for the National Cancer Institute includes the entire state of Indiana. Following HIPAA authorization from respondents to access their electronic health records, cancer screening information obtained through the survey were matched with the participant’s longitudinal EHR data by referencing each patient’s first and last name, birthdate, and place of residence. The electronic information was obtained from the Indiana Network for Patient Care (INPC), which is the clinical data repository for IHIE, a community-wide HIE operating in central Indiana, with the support of the Regenstrief Institute. The INPC consists of clinical observations across five major hospital systems, public health departments (both state and county), and Indiana Medicaid [13, 14]. The study was approved by the IUPUI Institutional Review Board.

### Study cohort

From a list of 284,062 people seen at least once in the past 12 months in the statewide health system and living in one of 34 Indiana counties with higher cancer mortality rates, a random, stratified sample of 8,000 adults was selected. In stratifying the sample, rural geographic location and race were equally weighted. The initial goal was to sample 2,000 individuals from each of four strata (rural White, rural Black, urban White, urban Black); due to the small number of participants in rural Black, the remaining 2,000 were taken from the rural White strata, resulting in 4,000 individuals from both rural and urban areas. Twenty-one patients were excluded from the sample because their primary care providers declined to authorize their participation in the survey, thus resulting in 7,979 mailed surveys. Out of all mailed surveys, a total of 970 adults aged 18–75 years completed the survey, generating a 12% response rate. Younger adults were included in the sample so as to collect data on cervical cancer screening behavior; the upper age limit of the sample was set at 75 years because guidelines do not routinely recommend cancer screening after age 75 [15]. Out of these 970 respondents, a total of 711 individuals provided HIPAA authorization (73.3%), comprising our final study sample. The survey methodology has been described in more detail elsewhere [12].

### Populations eligible for cancer screening

The participants in our study were assessed on their cancer screening behavior for three types of cancer: colorectal, cervical, and breast. Screening guidelines from the U.S. Preventive Services Task Force (USPSTF) were used to determine the sample of survey respondents eligible for appropriate screening tests. For colorectal cancer, the eligible sample included men and women aged 50–75 years to receive a colonoscopy every ten years or receive a fecal immunochemical test (FIT)/stool test every year [16]. For cervical cancer, the eligible sample included women aged 21–29 years to receive a Pap test every three years and 30–65 years to receive a Pap test every three years or a human papilloma virus (HPV) test every five years [17]. In the case of breast cancer, the eligible sample was taken from women aged 50–75 years to receive a mammography every two years [18].

### Survey-based cancer screening measures

When eligible for screening, the respondents were asked whether or not they reported receiving one of the three cancer screening approaches with responses being “Yes” or “No” (binary in nature). For colorectal cancer, patients were asked if they ever received a colonoscopy and whether they had one every ten years, as well as if they ever received a Fecal Immunochemical Test (FIT)/stool test, and whether they had one every year. For cervical cancer, patients were asked if they ever received a PAP test and if they had one every three years, or if they ever received a human papilloma virus (HPV) test and if they had one every five years. For breast cancer, the patients were asked whether they ever received a mammogram and if they had one every two years.

Respondents were also asked the time since their last screening test with responses being “Within the past year (less than 12 months ago)”, “More than 1 year ago, but less than 2 years ago”, “More than 2 years ago, but less than 3 years ago”, “More than 3 years ago, but less than 5 years ago”, or “5 or more years ago” (ordinal in nature). See Appendix 1 for detailed survey questions used for this study and Appendix 2 for the entire survey. Information on receipt of the screening and time since last screening were measured to assess the degree of concordance between survey self-report and HIE data.

## Statistical analysis

Descriptive statistics, adjusted for sampling weights, were performed of individual socio-demographic characteristics that are patient reported, including age, gender, race, educational level, marital status, insurance status, income, home ownership, employment status, rurality based on RUCA codes, and self-reported health status.

For the questions on receipt of cancer screening and time since last screening, as a first step, we conducted bivariate analysis on participants’ responses to the survey and the corresponding information in HIE data using Chi-square tests to check for any significant differences between the two information sources. As a second step, we evaluated the sensitivity, specificity (only for receipt of cancer screening), and concordance as validity measures between the two measures of screening information. For the questions on time since last screening, we only considered those participants whose HIE data as well as self-report indicated receipt of screening. All the analyses were adjusted for sampling weights.

To assess under-reporting, we estimated sensitivity (proportion of patients who self-reported having a screening test done among those with the test documented in their EMR). To assess over-reporting, we estimated specificity (proportion of patients who self-reported not having a screening test done among those without the test documented in their EMR). Finally, we used the Gwet’s agreement coefficient (Gwet’s AC) to measure the concordance of screening information obtained from HIE data and survey self-report. The Gwet's agreement coefficient (Gwet's AC), a measure of correlation, is defined as the conditional probability that two randomly chosen observational measurements will agree, assuming no agreement by chance. The agreement coefficients were calculated using Gwet's new chance-corrected inter-rater agreement coefficients weighted ordinally, extending all existing agreement coefficients to include multiple raters, multiple rating categories, any measurement level, and multiple ratings per subject.

As an additional analysis, we showed comparison between three different agreement measures—Gwet’s Agreement Coefficient, Fleiss Kappa and Intraclass Correlation Coefficient (ICC) (see Appendix 3) but we chose Gwet’s AC as the final measure of concordance for this study over the alternative measures^1^ for various statistical concerns [19–26]. The Gwet's AC is interpreted according to Landis and Koch's guidelines [27, 28].

The analyses were performed in Stata (Stata 16.1, StataCorp LLC, College Station, TX).

## Results

### Weighted descriptive statistics

Of the 711 patients surveyed, the participants were most often between ages 50–64 years(36%), female (63%), white (86%), partnered (60%), homeowners (69%), insured (96%), employed (47%), urban (89%), and reported very good general health (37%) (Table 1).

**Table 1:** Weighted summary statistics of participants’ sociodemographic variables

Patient Characteristics	Unweighted frequency (unweighted %)	Weighted frequency (weighted %)
	Total number of observations: 711	Total number of observations: 711; Population size: 27,271.102
**Age**		
18–34	84(11.81)	117(16.53)
35–49	102(14.35)	99(13.87)
50–64	272(38.26)	257(36.21)
65 +	253(35.58)	237(33.39)
**Sex**		
Male	325(45.71)	260(36.56)
Female	386(54.29)	451(63.44)
**Race**		
White	554(77.92)	612(86.08)
Black	131(18.42)	73(10.32)
Multi/Other	26(3.66)	26(3.60)
**Education**		
< High school	46(6.75)	39(5.78)
High school graduate (or GED)	173(25.40)	130(19.10)
Post HS/some college	189(27.75)	167(24.53)
College graduate or higher	273(40.09)	344(50.58)
**Marital Status**		
Partnered	434(62.72)	419(60.52)
Not partnered	258(37.28)	273(39.48)
**Health Insurance**		
Yes	658(94.54)	671(96.44)
No	38(5.46)	25(3.56)
**Income**		
$0–19,999	121(18.47)	116(17.72)
$20,000–49,999	203(30.99)	156(23.75)
$50,000–99,999	210(32.06)	193(29.55)
$100,000 +	121(18.47)	190(28.98)
**Own Home**		
Own	488(70.72)	475(68.81)
Rent/Occupy	202(29.28)	215(31.19)
**Employed**		
Yes	275(41.35)	314(47.27)
No	162(24.36)	149(22.31)
Retired	228(34.29)	202(30.42)
**Metro Status (based on RUCA codes)**		
Urban	329(46.27)	636(89.51)
Rural	382(53.73)	75(10.49)
**Self Reported Health**		
Excellent/Very good	215(30.32)	265(37.35)
Good	291(41.04)	257(36.25)
Fair/Poor	203(28.63)	187(26.39)

Results are listed as unweighted n (unweighted %) in column 2 and weighted n (weighted %) in column 3, with n being the sample size unless otherwise indicated. All the socio-demographic information is patient reported. 711 out of 970 patients (73.3%) who completed the survey, provided HIPAA authorization, allowing access to their electronic health information

### Weighted bivariate analysis

With regards to the receipt of screening, bivariate analysis showed statistically significant differences between the two data sources (survey self-report (SR) and EHR from IHIE) for all screening tests (*p*-value < 0.01) (Table 2, columns 2 and 3). The participants who reported positive receipt of cancer screening were also asked about their time since last screening. Bivariate analysis showed statistically significant differences between the two information sources for colonoscopy, Pap test, and mammogram (Table 3, columns 1 and 2).

**Table 2:** Summary validity measures of information on receipt of screening in survey self-report and IHIE (Weighted)

	**Survey responses (SR) (1)**	**EMR = No (2)**	**EMR = Yes (3)**	**Sensitivity (95% CI)** $\frac{True\;positive}{True\;positive+False\;negative}$ **(4)**	**Specificity (95% CI)** $\frac{True\;negative}{False\;positive+True\;negative}$ **(5)**	**Concordance- Gwet’s AC (95% CI)** ***Benchmark scale for reliability*** **(6)**
**Screening Tests**						
** Colonoscopy**	**SR = No** **SR = Yes**	45/505(8.91) 115/505(22.77)	39/505(7.72) 305/505(60.39)	$\frac{305}{305+39}=0.88$ (0.85,0.92)	$\frac{45}{115+45}=0.28$ (0.21,0.35)	0.52^a^ (0.44,0.59) *Moderate*
** FIT test**	**SR = No** **SR = Yes**	371/504(73.61) 19/504(3.77)	98/504(19.44) 15/504(2.98)	$\frac{15}{15+98}=0.13$ (0.07,0.21)	$\frac{371}{19+371}=0.95$ (0.93,0.97)	0.69^a^ (0.63,0.75) *Substantial*
** HPV test**	**SR = No** **SR = Yes**	64/161(39.75) 20/161(12.42)	43/161(26.71) 33/161(20.49)	$\frac{33}{33+43}=0.43$ (0.32,0.55)	$\frac{64}{20+64}=0.76$ (0.66,0.85)	0.24^a^ (0.08,0.40) *Fair*
** PAP test**	**SR = No** **SR = Yes**	22/185(11.89) 35/185(18.92)	24/185(12.97) 103/185(55.67)	$\frac{103}{103+34}=0.81$ (0.73,0.88)	$\frac{22}{35+22}=0.39$ (0.26,0.52)	0.46^a^ (0.33,0.60) *Moderate*
** Mammogram**	**SR = No** **SR = Yes**	14/255(5.49) 26/255(10.19)	24/255(9.41) 190/255(74.51)	$\frac{190}{190+24}=0.89$ (0.84,0.93)	$\frac{14}{26+14}=0.35$ (0.21,0.52)	0.73^a^ (0.65,0.81) *Substantial*

Results in columns 2 and 3 are listed as marginal totals and are statistically significant at 1% level of significance. Study sample is restricted to 711 out of 970 patients (73.3%) completing the survey, who provided HIPAA authorization, allowing access to their electronic health information. Gwet’s probabilistic benchmarking method according to the Landis and Koch scale of reliability is: -1.0–0.0 (Poor), 0.0–0.2 (Slight), 0.2–0.4 (Fair), 0.4–0.6 (Moderate), 0.6–0.8 (Substantial), 0.8–1.0 (Almost Perfect) [23]. The measures in all the columns are adjusted for sampling weights

*Abbreviations*: *SR* Self-report, *EMR* Electronic Medical Records, *FIT* Fecal immunochemical test, *HPV* Human Papillomavirus, *CI* Confidence Interval, *AC* Agreement Coefficient

^a^denotes significance at 1% level

^b^denotes significance at 5% level

^c^denotes significance at 10% level

**Table 3:** Agreement of information on time since last screening in survey self-report and IHIE (Weighted)

Screening Tests (Time since last screening)	Survey responses (1)	EMR (2)	Concordance- Gwet’s AC (95% CI) *Benchmark scale for reliability* (3)
**Colonoscopy** *n* = 220 (SR = 1 & EMR = 1)			0.53^a^ (0.43,0.63) *Moderate*
< 1 yr	60/220(27.27)	73/220(33.18)	
> 1 yr- < 2yrs	45/220(20.45)	41/220(18.64)	
> 2yrs- < 3yrs	35/220(15.91)	35/220(15.91)	
> 3yrs- < 5yrs	38/220(17.27)	24/220(10.91)	
> 5yrs- < 10yrs	42/220(19.09)	38/220(17.27)	
> = 10yrs	-	9/220(4.09)	
**FIT test** *n* = 11 (SR = 1 & EMR = 1)			0.21 (-0.21,0.64) *Fair*
< 1 yr	11/11(100.0)	4/11(36.36)	
> 1 yr- < 2yrs	-	2/11(18.18)	
> 2yrs- < 3yrs	-	2/11(18.18)	
> 3yrs- < 5yrs	-	2/11(18.18)	
> 5yrs- < 10yrs	-	-	
> = 10yrs	-	1/11(9.09)	
**HPV test** *n* = 26 (SR = 1 & EMR = 1)			0.48^a^ (0.21,0.75) *Moderate*
< 1 yr	9/26(34.61)	8/26(30.77)	
> 1 yr- < 2yrs	9/26(34.61)	12/26(46.15)	
> 2yrs- < 3yrs	3/26(11.54)	2/26(7.69)	
> 3yrs- < 5yrs	5/26(19.23)	8/26(30.77)	
> = 5yrs	-	-	
**Pap test** *n* = 84 (SR = 1 & EMR = 1)			0.58^a^ (0.44,0.72) *Moderate*
< 1 yr	36/84(42.86)	36/84(42.86)	
> 1 yr- < 2yrs	34/84(40.48)	19/84(22.62)	
> 2yrs- < 3yrs	14/84(16.67)	11/84(13.09)	
> 3yrs- < 5yrs	-	8/84(9.52)	
> = 5yrs	-	10/84(11.90)	
**Mammogram** *n* = 157 (SR = 1 & EMR = 1)			0.90^a^ (0.86,0.95) *Almost perfect*
< 1 yr	119/157(75.80)	134/157(85.35)	
> 1 yr- < 2yrs	38/157(24.20)	6/157(3.82)	
> 2yrs- < 3yrs	-	8/157(5.09)	
> 3yrs- < 5yrs	-	2/157(1.27)	
> = 5yrs	-	7/157(4.46)	

For the questions on time since last screening, the sample study participants are restricted to those whose HIE data as well self-report indicated receipt of screening. Results in columns 1 and 2 are listed as marginal totals and are statistically significant at 1% level of significance for Colonoscopy, Pap test and Mammogram. Gwet’s probabilistic benchmarking method according to the Landis and Koch scale of reliability is: -1.0–0.0 (Poor), 0.0–0.2 (Slight), 0.2–0.4 (Fair), 0.4–0.6 (Moderate), 0.6–0.8 (Substantial), 0.8–1.0 (Almost Perfect) [23]. The measures in all the columns are adjusted for sampling weights

*Abbreviations*: *SR* Self-report, *EMR* Electronic Medical Records, *FIT* Fecal immunochemical test, *HPV* Human Papillomavirus, *CI* Confidence Interval, *AC* Agreement Coefficient

^a^denotes significance at 1% level

^b^denotes significance at 5% level

^c^denotes significance at 10% level

The proportion of patients for whom both the HIE and self-report data indicated receipt of screening showed the following pattern: colonoscopy (305/505 = 60%), FIT test (15/504 = 3%), HPV test (33/161 = 20%), Pap test (103/185 = 56%), and mammogram (190/255 = 74%). Comparing the proportion of patients whose HIE data indicated screening (but self-report did not) with the proportion of patients whose self-report indicated screening (but HIE did not), the following patterns emerged: colonoscopy (8% HIE alone vs. 23% SR alone), FIT test (19% HIE alone vs. 4% SR alone), HPV test (27% HIE alone vs. 12% SR alone), Pap test (13% HIE alone vs. 19% SR alone), mammography (9% HIE alone vs. 10% SR alone) (Table 2).

### Weighted sensitivity, specificity and concordance (Receipt of cancer screening)

For receipt of cancer screening, patients’ self-reports showed high sensitivity with their corresponding information recorded in their EMRs for colonoscopy (sensitivity = 88%, 95% CI: 0.85–0.92), Pap test (sensitivity = 81%, 95% CI: 0.73–0.88) and mammogram (sensitivity = 89%, 95% CI: 0.84–0.93), thus indicating less under-reporting for these tests. However, for FIT (sensitivity = 13%, 95% CI: 0.07–0.21) and HPV tests (sensitivity = 43%, 95% CI: 0.32–0.55), patients’ self-reports showed low sensitivity and high specificity, indicating more under-reporting than over-reporting (Table 2, columns 4 and 5), With regards to the level of concordance of information on receipt of cancer screening between HIE data and survey self-report, Gwet’s AC showed the highest level of concordance for Mammogram (Gwet’s AC: 0.73, 95% CI: 0.65–0.81) and the lowest level of agreement for HPV test (Gwet’s AC: 0.24, 95% CI: 0.08–0.40).

To summarize, there was high sensitivity between the two information sources for colonoscopy, Pap test and mammogram, which are all procedures and low sensitivity for FIT and HPV tests, both laboratory tests. Screening receipt information from HIE data and survey-self report showed overall concordance ranging from 24 to 73%, indicating fair to substantial concordance [19] according to Gwet’s AC (Table 2, column 6).

### Weighted concordance (Time since last cancer screening)

For time since last screening, Gwet’s AC showed the highest level of agreement for mammogram timing (Gwet’s AC: 0.90, 95% CI: 0.86, 0.95) and the lowest level of agreement for FIT test timing (Gwet’s AC: 0.21, 95% CI: -0.21, 0.64 (although *p*-value > 0.10)), thus indicating almost perfect to fair concordance [19] according to Gwet’s AC (Table 3, column 3).

## Discussion

In our study we focused on the cancer screening phase and evaluated the concordance between HIE data and self-reported responses of surveyed Indiana residents seen in a statewide healthcare system for receipt of screenings and the time since receipt of the last screening test. For screening receipt, results indicated the highest level of agreement for mammogram and lowest level of agreement for HPV test. For screening timing, the highest level of agreement between the two data sources was for mammogram timing and the lowest level of agreement for FIT test timing. Additionally, HIE data provided relatively more information about FIT and HPV tests, which are both laboratory-based screening tests. Self-reported data provided more information about colonoscopy, Pap test, and mammography, all of which are medical screening procedures.

In the screening phase of the cancer care continuum, one of the earliest prior studies assessed the concordance between self-report and non-electronic medical record documentation among Kaiser Permanente Medical Care Program participants. Data were collected on the reason and timing for Pap tests, mammograms, clinical breast exams, fecal occult blood tests (FIT tests), digital rectal examinations, and sigmoidoscopies. Researchers found that self-reported response and non-electronic medical record documentation generally agreed more for procedures involving a test report (mammogram, Pap test, fecal occult blood test, and sigmoidoscopy) than a physician's note (clinical breast examination and digital rectal examination) [6]. The results of our study are similar to their findings, especially for mammogram timing and receipt of FIT test where we found the most agreement between HIE data and self-report. A relatively recent study conducted among patients in 25 New Jersey Primary Care Practices who participated in the SCOPE program (supporting colorectal cancer outcomes through participatory engagement) indicated evidence of agreement for cancer screening ranging from 61% for Pap and PSA test to 83% for colorectal endoscopy. In this study, self-reports had a higher rate than non-electronic medical records [7]. Both of these studies compared patients' self-reports against non-electronic medical records. Paper-based documentation may not provide accurate information on screening histories if information is entered incompletely; further, non-electronic medical records also suffer from disorganization, non-integration with other electronic systems, and lack of backups and security issues [29, 30]. Hence, our study has the advantage of using EHRs from HIE over non-electronic health records which is more consistent with current medical practice. Moreover, EHRs arguably provide higher quality data with more accessible, accurate, complete and up-to-date patient records with built-in privacy and security features [31, 32].

In the treatment phase of the cancer care continuum, an academic hospital cancer registry study among breast cancer survivors from 2004–2009 evaluated concordance first between electronic query and manual review used to extract EHR data, and second between survivors' self-reports and the extracted EHR data on post-treatment mammography. Electronic query identified more mammograms post-treatment than manual review, with high concordance between the two methods (0.90). Fewer days since mammogram were associated with better concordance between self-reporting and EHR data. In conclusion, Tiro et al. encouraged the use of self-report as a screening tool among cancer survivors for surveillance care delivery [10]. The advantage of our approach over prior EHR-based studies is the fact that they were studies performed within a single health care setting. On the other hand, EHRs in a state-based HIE, as explored in this study, use clinical data from among patient populations aggregated across multiple health care organizations [33]. In addition to offering a complete, accurate, and holistic view of patient records, HIEs reduce duplication of information on procedures or tests, improving the usefulness of patient health records. HIE also improves the accessibility of medical data across multiple clinical settings, thereby improving the capacity to use population data for public health purposes and other quality improvement activities across consortia of health care organizations [34–36]. Hence, using the Indiana Network of Patient Care (INPC), encompassing a community wide HIE, enables the measurement of population-based cancer screening behavior at a population level with greater efficiency and completeness.

## Limitations

Some study limitations must be considered when interpreting these results. First, our survey response rate was relatively low, at 12%, despite using established methods for survey research. Due to our expectation of a low response rate based upon current survey experience [37–39], enough surveys were delivered, with follow-up postcard reminders and a second copy of the survey to have meaningful population-based estimates. This was to ensure a relatively large absolute number of surveys among the target population. Other data collection methods, such as in-person interviews, might have improved our participation rate but would not have had the same reach as the mailed survey. Nonetheless, we received completed surveys from every surveyed county [40], and respondents and non-respondents did not differ significantly across available sociodemographic characteristics [41]. Second, we selected only those residents who had been involved with a single health system in Indiana, although IU Health is Indiana's largest integrated health system serving approximately 1 million individuals community wide https://iuhealth.org. Nonetheless, the results of our study should be interpreted as a sample from a statewide health system with at least some access to healthcare, as opposed to a population-based state sample. Overall, the access to healthcare by participants increased the likelihood of cancer screening receipt occurring. Finally, some HIEs have greater challenges to data sharing because of state-level variation in patient consent policies for sharing of health data [42]. Specifically, opt-in policies that require providers to consent each patient to sharing information with HIE programs increase administrative costs that make HIE more burdensome. Thus, all researchers will not have uniform access to HIE in their communities.

## Conclusion

Different data sources yielded different information value about the receipt of cancer screening, depending on the type of cancer screening. The HIE data, for example, provided relatively more information about FIT and HPV tests, both laboratory tests, than about colonoscopy, Pap tests, or mammograms, all procedures. To choose the ideal data collection method, studies that use a single data source should consider the type of cancer screening test. Both HIE and self-reports provided unique information about cancer screening. The most robust measurement approach involves collecting both HIE and self-reported screening information. When there are disagreements between the data sources, a practical approach may be to consider most positives measures of cancer screening tests as true positives, in order to overcome the risks of false negatives posed by HIE (missing data) and self-report (recall bias). If one source of data is used over another, it will likely create biases in prediction, for example algorithms based upon different sources of cancer screening data (HIE vs. self-reported) will very likely provide different predictions about cancer mortality, and furthermore, these predictions will vary by different race/ethnicity groups. Moreover, the optimal data source may vary depending on the outcome of interest being measured, whether it is clinical decision making, performance measurement, or population surveillance. Future research opportunities include looking at concordance between self-report and EHR data over time, concentrating on vulnerable populations. Data should also be considered from different EHR systems such as single vendor EHRs, as well as EHRs controlled by patients known as personal health records, to draw comparisons with corresponding patient self-report regarding cancer screening.

## Supplementary Information

**Additional file 1:** **Appendix 1.** Survey questions used for this research.

**Additional file 2. **

**Additional file 3:** **Appendix 3.** Comparison of different agreement measures.

## Data Availability

The datasets generated during and/or analyzed during the current study are not publicly available because public posting of database was not approved by the IUPUI IRB. However, datasets are available from the corresponding author on reasonable request.

## Abbreviations

EHR: Electronic Health Record
FIT: Fecal Immunochemical Test
Gwet’s AC: Gwet’s Agreement Coefficient
HIE: Health Information Exchange
HIPAA: Health Insurance Portability and Accountability Act
HPV: Human Papilloma Virus
IHIE: Indiana Health Information Exchange
INPC: Indiana Network for Patient Care
IU: Indiana University
IUPUI: Indiana University Purdue University
SCOPE: Supporting Colorectal Cancer Outcomes Through Participatory Engagement
SR: Self-report
USPSTF: United States Preventive Services Task Force

## Footnotes

1 Fleiss Kappa (κ), an extension of Cohen/Conger’s Kappa similar to correlation coefficients ranging from -1 to + 1 with 0 representing the amount of agreement expected from random choice, and 1 indicating perfect agreement is an alternative method to measure the level of concordance between raters. We choose Gwet’s AC over Fleiss Kappa as Kappa measures suffer from some statistical issues; it assumes independence between the raters, hence frequently generating agreement due to chance which is not entirely correct. However, the Gwet’s AC does not depend upon this assumption. Additionally, the Kappa values suffer from the “Kappa paradox”; they tend to change quite a lot with a change in prevalence, i.e., the values become high and close to the percentage agreement when there is high prevalence (the prevalence problem). Also, the degree to which observers disagree has an impact on the Kappa values (the bias problem). Gwet’s AC tends to lessen the kappa limitations; [19, 21] hence we consider Gwet’s AC providing more stable inter-rater reliability coefficients in our study following few recent studies that have also preferred Gwet’s AC over Kappa statistic or being a more stable inter-rater reliability coefficient [22–24]. Moreover, Fleiss Kappa is more appropriate to use when the number of raters (in our case, self-reports and HIE data) are more than two. ICC measure has limitations as well as it strongly depends on the population variance; ICC values tend to be higher when applied to more heterogeneous population in comparison with more homogenous populations, even when there are similar levels of agreement.
