# Supplementary material for: Comparison of health information exchange data with self-report in measuring cancer screening
Source: BMC Med Res Methodol. 2023 Jul 25;23:172. doi: 10.1186/s12874-023-01907-7 (PMC10367403; doi:10.1186/s12874-023-01907-7)
Supplement: Supplementary file 1 — Additional file 1: Appendix 1. Survey questions used for this research. [file 12874_2023_1907_MOESM1_ESM.docx]

**Appendix 1: Survey questions used for this research**

Below, we provided in detail the cancer screening related questions asked in the IUSCC Hoosier Health Survey.

For Colorectal Cancer

- Sigmoidoscopy and colonoscopy are exams in which a tube is inserted in the rectum to view the colon for signs of cancer or other health problems. Have you ever had either of these exams?
  1. Yes
  2. No
- For a SIGMOIDOSCOPY, a flexible tube is inserted into the rectum to look for problems. A COLONOSCOPY is similar, but uses a longer tube, and you are usually given medication through a needle in your arm to make you sleepy and told to have someone else drive you home after the test. Was your MOST RECENT exam a sigmoidoscopy or a colonoscopy?
  1. Yes
  2. No
- How long has it been since you had your last sigmoidoscopy or colonoscopy?
  1. Within the past year (less than 12 months ago)
  2. More than 1 year ago, but less than 2 years ago
  3. More than 2 years ago, but less than 3 years ago
  4. More than 3 years ago, but less than 5 years ago
  5. More than 5 years ago, but less than 10 years ago
  6. 10 or more years ago
- A blood stool test is a test that may use a special kit at home to determine whether the stool contains blood. Have you ever had this test using a home kit?
  1. Yes
  2. No
- How long has it been since you had your last blood stool test using a home kit?
  1. Within the past year (less than 12 months ago)
  2. More than 1 year ago, but less than 2 years ago
  3. More than 2 years ago, but less than 3 years ago
  4. More than 3 years ago, but less than 5 years ago
  5. 5 or more years ago

For Cervical Cancer

- A Pap test is a test for cancer of the cervix. Have you ever had a Pap test?
  1. Yes
  2. No
- How long has it been since you had your last Pap test?
  1. Within the past year (less than 12 months ago)
  2. More than 1 year ago, but less than 2 years ago
  3. More than 2 years ago, but less than 3 years ago
  4. More than 3 years ago, but less than 5 years ago
  5. 5 or more years ago
- An HPV test is sometimes given with the Pap test for cervical cancer screening. Have you ever had a HPV test?
  1. Yes
  2. No
- How long has it been since you had your last HPV test?
  1. Within the past year (less than 12 months ago)
  2. More than 1 year ago, but less than 2 years ago
  3. More than 2 years ago, but less than 3 years ago
  4. More than 3 years ago, but less than 5 years ago
  5. 5 or more years ago

For Breast Cancer

- A mammogram is an x-ray of each breast to look for breast cancer. Have you ever had a mammogram?
  1. Yes
  2. No
- How long has it been since you had your last mammogram?
  1. Within the past year (less than 12 months ago)
  2. More than 1 year ago, but less than 2 years ago
  3. More than 2 years ago, but less than 3 years ago
  4. More than 3 years ago, but less than 5 years ago
  5. 5 or more years ago
