# Supplementary material for: Comparison of health information exchange data with self-report in measuring cancer screening
Source: BMC Med Res Methodol. 2023 Jul 25;23:172. doi: 10.1186/s12874-023-01907-7 (PMC10367403; doi:10.1186/s12874-023-01907-7)
Supplement: Supplementary file 2 — Additional file 2. [file 12874_2023_1907_MOESM2_ESM.pdf]

INDIANA UNIVERSITY
SCHOOL OF MEDICINE

INDIANA UNIVERSITY
MELVIN AND BREN SIMON CANCER CENTER

INDIANA UNIVERSITY
SCHOOL OF NURSING

Hoosier Health Survey

- **What is this survey about?** This survey includes questions about your knowledge, beliefs, and habits regarding health information, healthy lifestyles, and ways to prevent cancer and detect it early.
- **Instructions:** Please mark your answers clearly in the boxes using pencil or dark pen. Examples: ■ ☒ ☐

We value your responses. Thank you very much for your help!

---

1 In general, would you say your health is...

☐ Excellent
☐ Very good
☐ Good
☐ Fair
☐ Poor

2 Have you ever looked for information about health or medical topics from any source?

☐ Yes
☐ No **[If NO, go to question 5]**

3 Think about the most recent time that you looked for information about health or a medical topic. Where did you go to first? (MARK ONLY ONE)

☐ Books
☐ Brochures, pamphlets, etc.
☐ Cancer organization
☐ Family
☐ Friend/Coworker
☐ Doctor or health care provider
☐ Internet
☐ Library
☐ Magazines
☐ Newspapers
☐ Telephone information number
☐ Complementary, alternative, or unconventional practitioner
☐ Social media site, such as Facebook, PatientsLikeMe, CaringBridge, etc.
☐ Other, please specify:

4 Thinking back to your most recent search for information about health or medical topics, how much do you agree or disagree with each of the following statements?

a. It took a lot of effort to get the information you needed.

Strongly agree	Somewhat agree	Somewhat disagree	Strongly disagree
☐	☐	☐	☐

b. You felt frustrated during your search for information.

Strongly agree	Somewhat agree	Somewhat disagree	Strongly disagree
☐	☐	☐	☐

c. You were concerned about the quality of the information.

Strongly agree	Somewhat agree	Somewhat disagree	Strongly disagree
☐	☐	☐	☐

d. The information you found was hard to understand.

Strongly agree	Somewhat agree	Somewhat disagree	Strongly disagree
☐	☐	☐	☐

5 In general, how much do you trust information about health or medical topics from each of the following sources?

	Not at all	A little	Some	A lot
A doctor	☐	☐	☐	☐
Family or friends	☐	☐	☐	☐
Government health agencies	☐	☐	☐	☐
Newspapers or magazines	☐	☐	☐	☐
Television	☐	☐	☐	☐
The Internet or World Wide Web	☐	☐	☐	☐

6 **Do you ever go on-line to access the Internet or World Wide Web, or to send and receive e-mail?**

- ☐ Yes
- ☐ No **[If NO, go to question 8]**

7 **In the past 12 months, when you have used the Internet, did you access it through…**

	Yes	No
A regular dial-up telephone line?	☐	☐
Broadband such as DSL, cable or FiOS?	☐	☐
A cellular network (i.e., phone, 3G/4G)?	☐	☐
A wireless network (Wi-Fi)?	☐	☐

8 **Please indicate if you have any of the following:**

	Yes	No
Tablet computer like an iPad, Samsung Galaxy, Motorola Xoom or Kindle fire	☐	☐
Smartphone, such as an iPhone, Android, Blackberry, or Windows phone	☐	☐
Basic cell phone only	☐	☐

9 **In the past 12 months, have you used the following ways to communicate with your doctor or a doctor's office?**

	Yes	No
Email	☐	☐
Electronic health record messaging systems like MyIUHealth	☐	☐
Text message	☐	☐
Facebook or other social media sites	☐	☐
Skype, FaceTime, or other video conference systems	☐	☐

10 **In the past 12 months, have you talked to a doctor, nurse, or other health professional about the following scenarios?**

	Yes	No
Any kind of health information you have gotten from the Internet	☐	☐
Where or how to find reliable health information (from the Internet)	☐	☐
Communicating with each other electronically (e.g. emailing or texting)	☐	☐

11 **Overall, how confident are you that you could get advice or information *about cancer* if you needed it?**

- ☐ Completely confident
- ☐ Very confident
- ☐ Somewhat confident
- ☐ A little confident
- ☐ Not confident at all

12 **In the past 12 months, have you used the Internet to look for information *about cancer* for yourself or a family member?**

- ☐ Yes
- ☐ No **[If NO, go to question 14]**

13 **How useful was the cancer-related information you got from the Internet?** (MARK ONLY ONE)

- ☐ Very useful
- ☐ Useful
- ☐ Moderately useful
- ☐ Not useful at all

14 **In a typical week, outside of your job or work around the house, how many days do you do any physical activity or exercise of at least moderate intensity, such as brisk walking, bicycling at a regular pace, and swimming at a regular pace?** (MARK ONLY ONE)

☐	☐	☐	☐	☐	☐	☐	☐
None	1	2	3	4	5	6	7 days per week

15 **In the past 12 months, have you used a computer, smartphone, or other electronic means to do any of the following?** (Mark all that apply for each statement)

	Yes	No, I didn't need this service	No, my doctor doesn't offer this service	No, this is too complicated to do	No, I'm not comfortable sharing my health information
Looked for your personal health information from your medical record	☐	☐	☐	☐	☐
Looked for personal health information for someone else's medical record	☐	☐	☐	☐	☐
Looked up test results	☐	☐	☐	☐	☐
Requested a medication refill from your doctor	☐	☐	☐	☐	☐

16 **Answer these questions thinking about YOUR neighborhood or community. Do you agree or disagree with these statements?** (Mark the BEST answer for each statement)

	Strongly agree	Somewhat agree	Neither agree or disagree	Somewhat disagree	Strongly disagree
People around here are willing to help their neighbors.	☐	☐	☐	☐	☐
This is a close-knit neighborhood or community.	☐	☐	☐	☐	☐
People in this neighborhood or community can be trusted.	☐	☐	☐	☐	☐
People in this neighborhood or community generally do not get along with each other.	☐	☐	☐	☐	☐
People in this neighborhood or community do not share the same values.	☐	☐	☐	☐	☐

17 **The questions in this section ask you about your community and where you live.** (Mark the BEST answer for each question)

	Not at all	Barely	A little	About half	Pretty much	Very much	Extremely
How much do you see yourself belonging to a rural community?	☐	☐	☐	☐	☐	☐	☐
How much is being from a rural community a part of who you are?	☐	☐	☐	☐	☐	☐	☐
How much do you identify with people who live in rural communities?	☐	☐	☐	☐	☐	☐	☐
To what extent do you feel your general attitudes and opinions are similar to people who live in rural communities?	☐	☐	☐	☐	☐	☐	☐
To what extent do you feel that you are typical of people who live in rural communities?	☐	☐	☐	☐	☐	☐	☐
To what extent do you consider yourself a "city" person?	☐	☐	☐	☐	☐	☐	☐

18 **Have you ever been diagnosed as having cancer?**

☐ Yes ☐ No **[If NO, go to question 21]**

19 **What type of cancer did you have?**

☐

20 **At what age were you first told that you had cancer?**

☐☐

CANCER SCREENING

21 **A blood stool test is a test that may use a special kit at home to determine whether the stool contains blood. Have you ever had this test using a home kit?**

☐ Yes

☐ No **[If NO, go to question 23]**

22 **How long has it been since you had your last blood stool test using a home kit?**

☐ Within the past year (less than 12 months ago)

☐ More than 1 year ago, but less than 2 years ago

☐ More than 2 years ago, but less than 3 years ago

☐ More than 3 years ago, but less than 5 years ago

☐ 5 or more years ago

23 **Sigmoidoscopy and colonoscopy are exams in which a tube is inserted in the rectum to view the colon for signs of cancer or other health problems. Have you ever had either of these exams?**

☐ Yes

☐ No **[If NO, go to question 26]**

For a SIGMOIDOSCOPY, a flexible tube is inserted into the rectum to look for problems. A COLONOSCOPY is similar, but uses a longer tube, and you are usually given medication through a needle in your arm to make you sleepy and told to have someone else drive you home after the test.

24 **Was your MOST RECENT exam a sigmoidoscopy or a colonoscopy?**

☐ Sigmoidoscopy ☐ Colonoscopy

25 **How long has it been since you had your last sigmoidoscopy or colonoscopy?**

☐ Within the past year (less than 12 months ago)

☐ More than 1 year ago, but less than 2 years ago

☐ More than 2 years ago, but less than 3 years ago

☐ More than 3 years ago, but less than 5 years ago

☐ More than 5 years ago, but less than 10 years ago

☐ 10 or more years ago

26 **Have you ever had a lung scan, also called a low-dose CT scan, to screen for lung cancer?**

☐ Yes

☐ No **[If NO, go to question 28]**

27 **How long has it been since you had your last lung scan?**

☐ Within the past year (less than 12 months ago)

☐ More than 1 year ago, but less than 2 years ago

☐ More than 2 years ago, but less than 3 years ago

☐ 3 or more years ago

28 **A vaccine to prevent the human papillomavirus or HPV infection is available and is called the cervical cancer or genital warts vaccine, HPV shot, GARDASIL or CERVARIX. Have you EVER had an HPV vaccination?**

☐ Yes → **How many HPV shots did you receive?** ☐

☐ No

☐ Doctor refused when asked

The next questions are about screening for BREAST and CERVICAL cancer. If you are FEMALE, please answer questions 29 through 35. If you are MALE, go to question 36.

29 **A mammogram is an x-ray of each breast to look for breast cancer. Have you ever had a mammogram?**

☐ Yes

☐ No **[If NO, go to question 31]**

30 **How long has it been since you had your last mammogram?**

☐ Within the past year (less than 12 months ago)

☐ More than 1 year ago, but less than 2 years ago

☐ More than 2 years ago, but less than 3 years ago

☐ More than 3 years ago, but less than 5 years ago

☐ 5 or more years ago

31 **A Pap test is a test for cancer of the cervix. Have you ever had a Pap test?**

- ☐ Yes
- ☐ No **[If NO, go to question 33]**

32 **How long has it been since you had your last Pap test?**

- ☐ Within the past year (less than 12 months ago)
- ☐ More than 1 year ago, but less than 2 years ago
- ☐ More than 2 years ago, but less than 3 years ago
- ☐ More than 3 years ago, but less than 5 years ago
- ☐ 5 or more years ago

33 **Have you had a hysterectomy?**

- ☐ Yes
- ☐ No

34 **An HPV test is sometimes given with the Pap test for cervical cancer screening. Have you ever had an HPV test?**

- ☐ Yes
- ☐ No **[If NO, go to question 36]**

35 **How long has it been since you had your last HPV test?**

- ☐ Within the past year (less than 12 months ago)
- ☐ More than 1 year ago, but less than 2 years ago
- ☐ More than 2 years ago, but less than 3 years ago
- ☐ More than 3 years ago, but less than 5 years ago
- ☐ 5 or more years ago

36 **Compared to people your same age, how likely are you to get cancer in your lifetime?**

Very unlikely	Unlikely	Neither unlikely nor likely	Likely	Very likely
☐	☐	☐	☐	☐

37 **How much do you agree or disagree with each of the following statements?**

a. It seems like everything causes cancer.

Strongly agree	Somewhat agree	Somewhat disagree	Strongly disagree
☐	☐	☐	☐

b. There are so many different recommendations about preventing cancer, it's hard to know which ones to follow.

Strongly agree	Somewhat agree	Somewhat disagree	Strongly disagree
☐	☐	☐	☐

c. When I think about cancer, I automatically think about death.

Strongly agree	Somewhat agree	Somewhat disagree	Strongly disagree
☐	☐	☐	☐

d. There's not much you can do to lower your chances of getting cancer.

Strongly agree	Somewhat agree	Somewhat disagree	Strongly disagree
☐	☐	☐	☐

e. I'd rather not know my chance of getting cancer.

Strongly agree	Somewhat agree	Somewhat disagree	Strongly disagree
☐	☐	☐	☐

38 **How worried are you about getting cancer?**

- ☐ Not at all
- ☐ Slightly
- ☐ Somewhat
- ☐ Moderately
- ☐ Extremely

39 **At what age are most women supposed to start having mammograms?**

☐☐

40 **At what age are most people supposed to start doing home blood stool tests, having a sigmoidoscopy or having a colonoscopy?**

☐☐

41 **At what age should long-term cigarette smokers start having a lung cancer screening test?**

☐☐

42 **Have you ever smoked at least 100 cigarettes in your entire life?**

☐ Yes

☐ No **[If NO, go to question 47]**

43 **How many years did you smoke?**

☐☐

44 **When you smoked, how many cigarettes per day did you smoke?**

☐☐

45 **Do you now smoke cigarettes…**

☐ Every day

☐ Some days

☐ Not at all

46 **Have you tried to quit smoking in the past 12 months?**

☐ Yes ☐ No

47 **Do you regularly use:**

	Yes	No
E-cigarettes	☐	☐
Smokeless (chewing) tobacco	☐	☐
Waterpipe or Hookah	☐	☐

48 **Is there a place that you USUALLY go to when you are sick or need advice about your health?**

☐ Yes

☐ There is NO place

☐ There is MORE THAN ONE place

49 **What kind of place do you go most often?** (MARK ONLY ONE)

☐ Clinic or health center

☐ Doctors office or HMO

☐ Hospital emergency room

☐ Hospital outpatient department

☐ Some other place

☐ Don't go to one place most often

50 **Was there a time in the past 12 months when you needed to see a doctor, but could not because of cost?**

☐ Yes ☐ No

51 **Do you have any kind of health care coverage, including health insurance, prepaid plans such as HMOs, or government plans such as Medicare, or Indian Health Service?**

☐ Yes ☐ No

52 **In the past 12 months, have you received some or all of your health care from a VA hospital or clinic?**

☐ Yes ☐ No

53 **What is your age?**

☐☐

54 **Are you male or female?**

☐ Male ☐ Female

55 **What is the highest grade or level of schooling you completed?** (MARK ONLY ONE)

☐ Less than 8 years

☐ 8 through 11 years

☐ 12 years or completed high school (or GED)

☐ Post high school training other than college (vocational or technical)

☐ Some college

☐ College graduate

☐ Postgraduate

56 Do you currently own or rent your home?

- [ ] Own
- [ ] Rent
- [ ] Occupied without paying monetary rent

57 What is your marital status? (MARK ONLY ONE)

- [ ] Married
- [ ] Living as married
- [ ] Divorced
- [ ] Widowed
- [ ] Separated
- [ ] Single, never been married

58 Thinking about members of your family living in your household, what is your combined annual income, meaning the total pre-tax income from all sources earned in the past year? (MARK ONLY ONE)

- [ ] $0 to $9,999
- [ ] $10,000 to $14,999
- [ ] $15,000 to $19,999
- [ ] $20,000 to $34,999
- [ ] $35,000 to $49,999
- [ ] $50,000 to $74,999
- [ ] $75,000 to $99,999
- [ ] $100,000 to $199,999
- [ ] $200,000 or more

59 Which one of these comes closest to your own feelings about your household's income these days?

- [ ] Living comfortably on present income
- [ ] Getting by on present income
- [ ] Finding it difficult on present income
- [ ] Finding it very difficult on present income

60 What is your current occupational status? (MARK ONLY ONE)

- [ ] Employed
- [ ] Unemployed
- [ ] Homemaker
- [ ] Student
- [ ] Retired
- [ ] Disabled
- [ ] Other, please specify:

61 Were you born in the United States?

- [ ] Yes
- [ ] No

62 What is your race? (MARK ALL THAT APPLY)

- [ ] White
- [ ] Black or African American
- [ ] American Indian or Alaska Native
- [ ] Asian
- [ ] Native Hawaiian or Other Pacific Islander
- [ ] Other, please specify:

63 Are you Hispanic, Latino/a, or Spanish origin? (MARK ALL THAT APPLY)

- [ ] Mexican, Mexican American, Chicano/a
- [ ] Puerto Rican
- [ ] Cuban
- [ ] Another Hispanic, Latino/a, or Spanish origin
- [ ] None of these

64 Would you be willing to allow us to contact you in the future about other studies for which you may be interested?

- [ ] Yes
- [ ] No

Thank you very much for completing the survey!

Please use the enclosed postage-paid envelope to return your completed survey.

You may also mail it directly to:

Indiana University
Center for Survey Research
1900 E Tenth St 3-South
Bloomington, IN 47406-7512
