# Supplementary material for: Comparison of health information exchange data with self-report in measuring cancer screening
Source: BMC Med Res Methodol. 2023 Jul 25;23:172. doi: 10.1186/s12874-023-01907-7 (PMC10367403; doi:10.1186/s12874-023-01907-7)
Supplement: Supplementary file 3 — Additional file 3: Appendix 3. Comparison of different agreement measures. [file 12874_2023_1907_MOESM3_ESM.docx]

**Appendix 3: Comparison of different agreement measures**

| **Screening tests** | **Gwet’s AC**  **(95% CI)**  **Benchmark scale for reliability**  **(1)** | **Fleiss Kappa**  **(95% CI)**  **Benchmark scale for reliability**  **(2)** | **Intraclass correlation coefficient (ICC)**  **(95% CI)**  **Benchmark scale for reliability**  **(3)** |
| --- | --- | --- | --- |
| **Colonoscopy** |  |  |  |
| Receipt of screening | 0.52***  (0.44,0.59)  *Moderate* | 0.17***  (0.07,0.26)  *Slight* | 0.20***  (0.12,0.29)  *Poor* |
| Time since last screening | 0.53***  (0.43,0.63)  *Moderate* | 0.36***  (0.24,0.48)  *Fair* | 0.42***  (0.31,0.52)  *Poor* |
| **Fit test** |  |  |  |
| Receipt of screening | 0.69***  (0.63,0.75)  *Substantial* | 0.07  (-0.03,0.16)  *Slight* | 0.11***  (0.02,0.20)  *Poor* |
| Time since last screening | 0.21  (-0.21,0.64)  *Fair* | -0.27***  (-0.44,-0.10)  *Poor* | 0.00  (0.00,0.58)  *Poor* |
| **HPV test** |  |  |  |
| Receipt of screening | 0.24***  (0.08,0.40)  *Fair* | 0.18**  (0.03,0.34)  *Slight* | 0.22***  (0.06,0.36)  *Poor* |
| Time since last screening | 0.48***  (0.21,0.75)  *Moderate* | 0.28**  (0.03,0.52)  *Fair* | 0.38**  (0.00,0.66)  *Poor* |
| **PAP test** |  |  |  |
| Receipt of screening | 0.46***  (0.33,0.60)  *Moderate* | 0.20***  (0.05,0.36)  *Slight* | 0.24***  (0.10,0.37)  *Poor* |
| Time since last screening | 0.58***  (0.44,0.72)  *Moderate* | 0.19***  (0.05,0.33)  *Slight* | 0.34***  (0.14,0.52)  *Poor* |
| **Mammogram** |  |  |  |
| Receipt of screening | 0.73***  (0.65,0.81)  *Moderate* | 0.24***  (0.09,0.40)  *Fair* | 0.27***  (0.15,0.38)  *Poor* |
| Time since last screening | 0.90***  (0.86,0.95)  *Almost perfect* | 0.18***  (0.05,0.31)  *Slight* | 0.15**  (0.00,0.29)  *Poor* |

*Note:* Study sample is restricted to 711 out of 970 patients (73.3%) completing the survey, who provided HIPAA authorization, allowing access to their electronic health information. For the questions on time since last screening, the sample study participants are restricted to those whose HIE data as well self-report indicated receipt of screening. Gwet’s probabilistic benchmarking method according to the Landis and Koch scale of reliability is: -1.0-0.0 (Poor), 0.0-0.2 (Slight), 0.2-0.4 (Fair), 0.4-0.6 (Moderate), 0.6-0.8 (Substantial), 0.8-1.0 (Almost Perfect).[23] Fleiss Kappa interpretation according to Landis and Koch (1977) is: <0 (Poor agreement), 0.01-0.20 (Slight agreement), 0.21-0.40 (Fair agreement), 0.41-0.60 (Moderate agreement), 0.61-0.80 (Substantial agreement), 0.81-1.00 (Almost perfect agreement).[Landis, J. R., & Koch, G. G. (1977). The measurement of observer agreement for categorical data. *biometrics*, 159-174] Intraclass correlation coefficient (ICC) interpretation according to Koo and Li (2016) is: below 0.50 (Poor), between 0.50-0.75 (Moderate), between 0.75-0.90 (Good), above 0.90 (Excellent).[ Koo, T. K., & Li, M. Y. (2016). A guideline of selecting and reporting intraclass correlation coefficients for reliability research. *Journal of chiropractic medicine*, *15*(2), 155-163] The measures in all the columns are adjusted for sampling weights. *** denotes significance at 1% level; ** denotes significance at 5% level; * denotes significance at 10% level. Abbreviations: SR = Self-report; EMR = Electronic Medical Records; FIT = Fecal immunochemical test; HPV = Human Papillomavirus; CI = Confidence Interval; AC = Agreement Coefficient.
